# Diagnostic Performance of the Darth Vader Sign for the Diagnosis of Lumbar Spondylolysis in Routinely Acquired Abdominal CT

**DOI:** 10.3390/diagnostics13152616

**Published:** 2023-08-07

**Authors:** Florian A. Huber, Cynthia S. Schmidt, Hatem Alkadhi

**Affiliations:** Institute of Diagnostic and Interventional Radiology, University Hospital Zurich, University of Zurich, Raemistrasse 100, 8091 Zurich, Switzerlandcynthia.schmidt@hotmail.de (C.S.S.)

**Keywords:** computed tomography, spondylolisthesis, spondylolysis, lumbar spine

## Abstract

Spondylolysis is underdiagnosed and often missed in non-musculoskeletal abdominal CT imaging. Our aim was to assess the inter-reader agreement and diagnostic performance of a novel “Darth Vader sign” for the detection of spondylolysis in routine axial images. We performed a retrospective search in the institutional report archives through keyword strings for lumbar spondylolysis and spondylolisthesis. Abdominal CTs from 53 spondylolysis cases (41% female) and from controls (*n* = 6) without spine abnormalities were identified. A total of 139 single axial slices covering the lumbar spine (86 normal images, 40 with spondylolysis, 13 with degenerative spondylolisthesis without spondylolysis) were exported. Two radiology residents rated all images for the presence or absence of the “Darth Vader sign”. The diagnostic accuracy for both readers, as well as the inter-reader agreement, was calculated. The “Darth Vader sign” showed an inter-reader agreement of 0.77. Using the “Darth Vader sign”, spondylolysis was detected with a sensitivity and specificity of 65.0–88.2% and 96.2–99.0%, respectively. The “Darth Vader sign” shows excellent diagnostic performance at a substantial inter-reader agreement for the detection of spondylolysis. Using the “Darth Vader sign” in the CT reading routine may be an easy yet effective tool to improve the detection rate of spondylolysis in non-musculoskeletal cases and hence improve patient care.

## 1. Introduction

Throughout the past decades, possibly even since before the Battle of Yavin [1,2], efforts in improving the understanding of defects in the pars interarticularis of lumbar vertebrae have resulted in an imperial extent of the literature regarding pathophysiology, terminology, categorization, and classification of spondylolysis and spondylolisthesis among the radiologic and orthopedic community [3,4,5,6,7].

Spondylolysis, in general, is a common medical entity described as damaged bone in the bony part of vertebral bodies that forms a bilateral joint with the adjacent vertebrae, called the “pars interarticularis”. The first documentation is likely to have been done by the Belgian anatomist Vesalius who identified the pars and its anatomical feature in terms of anteroposterior stability of the spine. Since then, the small bony structure has been the subject of constant and ongoing discussion. Primarily, this discussion has focused on the pathophysiologic principles behind spondylolysis and spondylolisthesis, and subsequently, the correct terminology and categorization of different types of these disorders began long before the large-scale availability of medical imaging [3,4]. To date, there has been disagreement about the differentiation between congenital and developmental variants of lumbar spondylolysis and subsequent spondylolisthesis. Whereas the classification of spondylolytic spondylolisthesis by Wiltse, Newman, and Macnab from 1976 [5] is considered the most common, other approaches to classification are also being used. For example, the system proposed by Marchetti and Bartolozzi [6] is well known and has been emphasized in a more recent joint summary statement of The Scoliosis Research Society and a focus group of the “Spine” journal [7]. In contrast to many other classifications, the authors here avoid referring to “congenital” or “isthmic” spondylolisthesis. In addition to terminology discussions, different scales of severity of spondylolisthesis are also subject to scientific debates [8,9]. The most widely accepted one, however, does not differentiate between cases caused by spondylolysis (“spondylolisthesis vera”) and remainders. The so-called Meyerding classification defines the extent of spondylolisthesis by its translational displacement, using a semiquantitative approach (scale from 0 to 4) [10,11]. Current investigations also focus on the automated assessment of spondylolysis and anterior displacement [12,13].

With respect to imaging-related questions, early and recent work has largely focused on the diagnostic performance of modalities for the detection of spondylolysis, such as computed tomography (CT), single photon emission CT [14,15,16,17,18], and magnetic resonance imaging [19,20,21], in comparison with the orthopedic reference modality of upright lateral radiography with or without flexion-extension radiographs [22,23,24].

Additionally, a number of groups aimed to identify diagnostic main and auxiliary imaging features for the accurate description of visual findings in spondylolysis and spondylolisthesis. The first-time definition of cross-sectional findings in those patients dates to the early years of CT imaging. Most importantly, McAfee and Yuan performed a thorough case analysis in which a relevant benefit from CT imaging for surgical planning was discovered [25]. From high-resolution axial CT images the authors deduced that CT reliably reveals defects in the pars interarticularis, characterized by an osseous discontinuation of an irregular surface with adjacent osteosclerosis [25].

In addition to the studies of McAfee [25] and others [14], the continuously increasing availability of high-resolution cross-sectional imaging resulted in a presumably increasing prevalence of spondylolysis in the general population and in patients suffering from lower-back pain [26,27,28]. Thus, a series of investigations focused on the correlation between spondylolysis vs. spondylolisthesis and clinical symptoms [29,30,31]. Exemplarily, the relation with repetitive force due to occupation, e.g., in infantry soldiers with high axial load, is being discussed controversially [30,31]. To date, a causal relationship between lower-back or radicular pain and spondylolysis/spondylolisthesis is a matter of continuous debate [22].

In the author’s experience, musculoskeletal disease is often underreported when reading body CT for non-skeletal reasons. This concurs with observations on the underreporting of skeletal disorders in body imaging by other groups [32,33,34]. For example, Donald and Barnard investigated 241 body CT cases of diagnostic radiology errors and reported bone findings as the most common perceptual errors, accounting for 10% of all cases [32]. For improving the radiologists’ perception and interpretation, the use of multiplanar reformations is advocated, particularly for spine imaging [35,36]. Nonetheless, the implementation of such easy-to-use tools into routine diagnostic workflows varies across radiology institutions [34,37]. With regard to spondylolysis in specific, we believe that in non-skeletal CT readings, the detection rate may benefit from more appreciation by radiologists in their daily clinical routine. Moreover, increased awareness and subsequent reporting of spondylolysis in body CT reading may help in assessing the clinical relevance of this entity by adding more robust data with regard data to prevalence among asymptomatic patients.

Thus, our aim was to introduce and evaluate a new, easy-to-use sign that allows for recognizing spondylolysis during clinical non-skeletal CT reading routine.

## 2. Materials & Methods

### 2.1. Definition of the Darth Vader Sign

**Figure 1:**
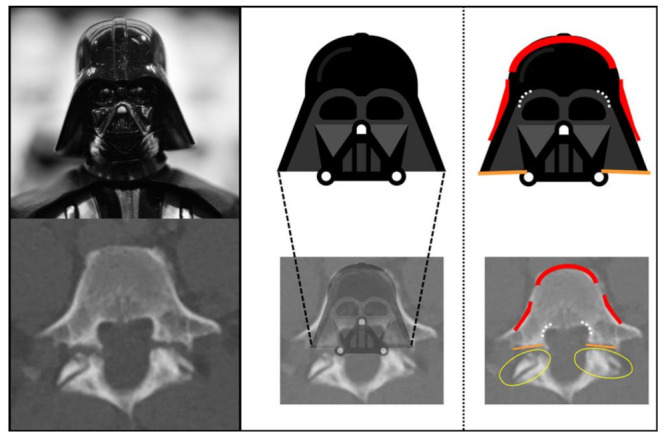
Typical appearance characteristics of Darth Vader and overlap with imaging features of spondylolysis in computed tomography.

Based on different described CT characteristics of lumbar spine spondylolysis (pars interarticularis defects, irregular osseous discontinuations, and osteosclerotic changes) [25], a mind-mapping process was used to find similarities in shape and proportions between axial images of the pathologic lumbar spine and commonly recognized logos, brands, and other elements suitable for a sign. In a lucky moment, we recognized major similarities to the face mask design of the lord Darth Vader (Figure 1).

Whereas the typical helmet of Darth Vader can be recognized as the shape of the anterior and anterolateral aspects of the lumbar vertebral body (all red), a simplified two-dimensional impression of Lord Darth Vader’s neck guard represents the irregular, flat-shaped discontinuation in the pars interarticularis (orange lines). In contrast, articular gaps can be clearly differentiated due to angle and surface texture. As additional auxiliary detail, the inner shape of the helmet at the orbital region can be imagined as the profound outline of the corpus–pedicle transition adjacent to the spinal canal (dotted white arches).

The criteria contributing to this diagnostic sign were identified as (i) a helmet-shaped anterior edge of the vertebral body, (ii) the presence of osseous interruptions not representing the facet joint, and (iii) comparably flat-shaped osseous interruptions with irregular margins. Cases where these osseous interruptions were present only on one side of the vertebra were considered as positive “Darth Vader sign” as well.

### 2.2. Feasibility Testing

**Figure 2:**
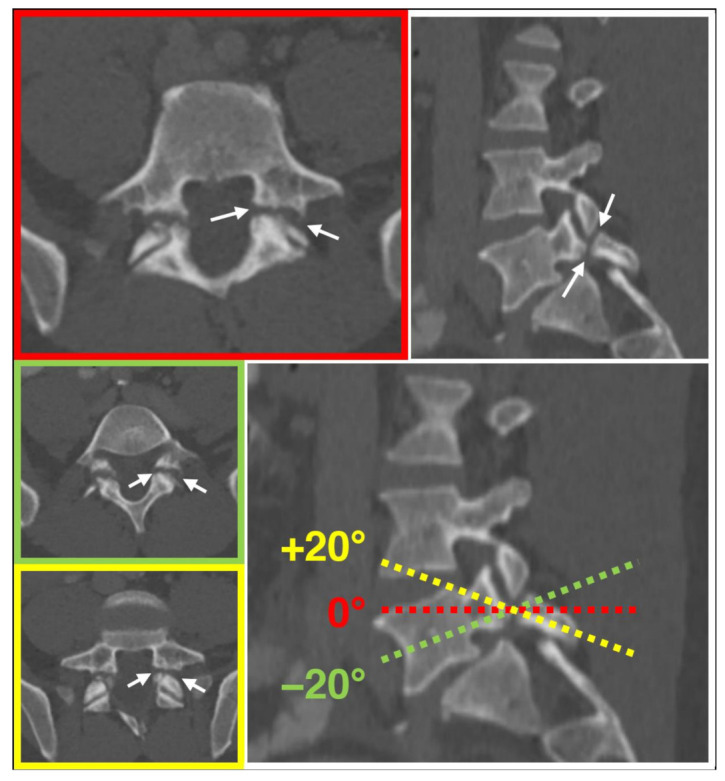
Independence of the “Darth Vader sign” from plane angulation. Defects of the pars interarticularis (arrows) and the respective criteria of the “Darth Vader sign” are noted in axial (red), as well as in axial oblique of +20 (yellow) and −20 (green) degrees.

**Figure 3:**
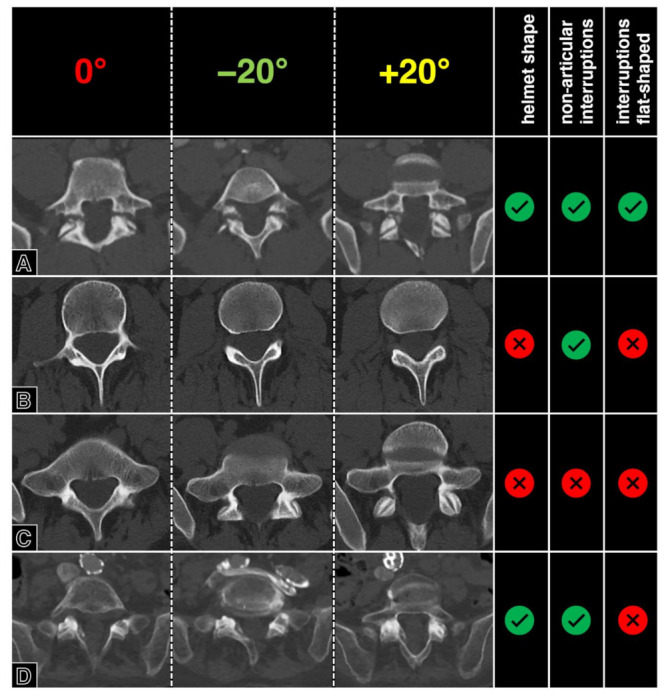
Presence of the “Darth Vader sign” criteria in cases with and without spondylolysis. From left to right, representative axial views at standard orientation, −20° tilt, and +20° tilt are being shown top to bottom for (**A**) a patient with bilateral spondylolysis, (**B**) a healthy individual with normal imaging of the third vertebral body, (**C**) a healthy individual with increased lumbar lordosis and marginal degeneration of the facet joints, and (**D**) from a patient with degenerative spondylolisthesis (Meyerding grade 1) due to severe facet joint osteoarthritis of the fifth lumbar vertebral body.

One resident radiologist (F.A.H.), supervised by an expert radiologist (H.A.), investigated the presence of the aforementioned three imaging features in one case each of “spondylolysis” and “degenerative spondylolisthesis without spondylolysis”, and on one healthy spine case. Furthermore, the presence of the three criteria was tested for dependency on angulations in one case. For this, a range of +/−20° oblique tilt reconstructions were visually analyzed for the presence of the “Darth Vader sign” criteria. Tilting the perpendicular plane of the z-axis showed no relevant impact on the presence of the criteria defining the “Darth Vader sign” (Figure 2), which was identified only in the “spondylolysis” case (Figure 3).

### 2.3. Patient and Image Selection

We then performed a retrospective analysis of text and CT image data from the radiology information and picture archiving and communication system (PACS) of the University Hospital Zurich in Switzerland, a 900-bed tertiary care hospital, including cases from May the fourth, 2017, until May the fourth, 2021.

A full-text search was used to identify reports from abdominal CT examinations that contained one of the following search strings in the free-text reporting field: “listh”, “spondylolys” (i.e., the respective word stems in German). The search function was limited to the Final Results sections of written reports and identified 65 examinations of concern. Of these initial 65 imaging studies, reports were excluded in cases of non-diagnostic image quality (*n* = 2), postoperative status of the spine (*n* = 2), and severely distorted alignment or geometry due to musculoskeletal disease (*n* = 8) by an expert radiologist (H.A.). Additionally, 6 random age-matched cases showing no spine abnormalities at CT were included as controls, forming a total of 59 imaging studies, i.e., one per patient. The controls were recruited retrospectively, considering examinations of the same inclusion period as spondylolysis cases. We used the hospital’s PACS system for the exclusion and selection of cases, including CT images from the power of the Force scanner (SOMATOM Force, dual-source CT, Siemens Healthineers, Germany). We included images only from dedicated examinations of the abdomen and pelvis, which were not requested due to musculoskeletal indications. Thus, the examinations had been performed for a variety of non-musculoskeletal clinical indications (staging, hemorrhage, infection, etc.), of which we did not further exclude any type of examination. Non-contrast and contrast-enhanced types were included. All of the included studies had comparable reconstruction parameters, the most commonly used being as follows: slice thickness, 2.0 mm; increment, 1.6 mm; Kernel Br36; pixel spacing variable due to different patient sizes; and contrast delay of 70 s in contrast-enhanced examinations.

Subsequently, all cases were reviewed by a senior consultant radiologist (H.A.) with 15 years of experience in imaging, which served as the reference standard. The expert had unrestricted access to image data and was allowed to use multiplanar reconstructions as needed. All cases were defined as either uni- or bilateral spondylolysis with or without spondylolisthesis (”spondylolysis”: *n* = 40, 6 of those unilateral; 14 female; mean age: 55.5 years), as degenerative spondylolisthesis without defects of the pars interarticularis (“degenerative spondylolisthesis”: *n* = 13; 8 female; mean age: 73.5 years), or as normal controls (“controls”: *n* = 6; 2 female; mean age: 54.8 years).

**Figure 4:**
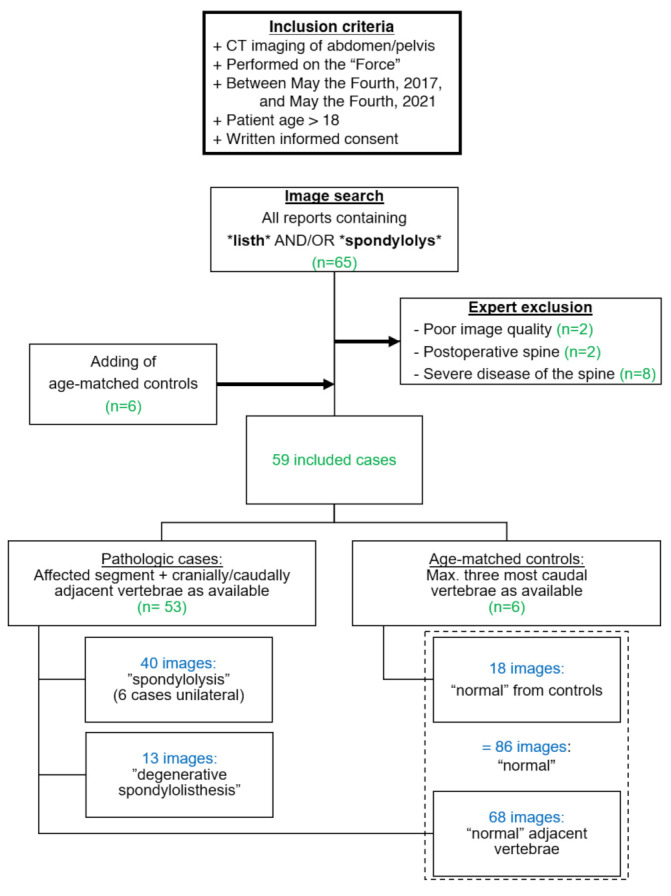
Workflow of included patients, according to the STARD guidelines.

Finally, one resident radiologist (F.A.H.) performed a slice-per-slice review of all axial image data and identified the single slice that best depicted the pars interarticularis of vertebral bodies affected by spondylolysis and/or anterolisthesis. Image export for that slice, as well as for slices of the cranially and, where applicable, caudally adjacent normal vertebrae in an analogous fashion, was performed. For the control group, analogous axial images were derived from the three most caudal vertebrae, as available (usually third to fifth lumbar vertebra; L3–L5). Image data was exported into a lossless file format without further postprocessing. This resulted in 40 images showing “spondylolysis” (of those, 6 cases were unilateral), 13 images showing “degenerative spondylolisthesis”, and 86 images showing no abnormalities (68 images as adjacent normal vertebrae from either “spondylolysis” or “degenerative spondylolisthesis” cases and 18 normal images from the control group). A STARD-compliant study flowchart is provided in Figure 4.

### 2.4. Image Readout

After randomization, we performed an independent, blinded image readout by two radiologists, one in their third (C.S.S., “R3”) and one in their fourth year (F.A.H., “R4”, two months delay after the image selection in order to avoid recall bias) of their residency program. The two readers had to decide whether or not they could identify the “Darth Vader sign” on the axial CT image according to the scheme presented in Figure 1 and as defined by the three image criteria defined above. A positive “Darth Vader sign” was rated only when all three underlying criteria were visually present to the reader.

### 2.5. Statistical Analysis

The results of the two independent readers were compared with the expert reference standard, and descriptive statistics were used to report the presence of the “Darth Vader sign” across the three different groups: “spondylolysis”, “degenerative spondylolisthesis”, and “controls”. Sensitivity and specificity were reported as a result of a contingency table. In addition, the area under the receiver operating characteristic curve (AUC) was used to calculate the diagnostic performance for each reader, separately from detecting the “Darth Vader sign” in either bilateral only or in all spondylolysis cases. Interpretation of the diagnostic accuracy was done according to Hosmer and Lemeshow [38].

We assessed the inter-reader agreement with Cohen’s Kappa and 95% confidence intervals. Level of agreement was interpreted according to Landis and Koch [39]. Age differences between the three study groups (“spondylolysis”, “degenerative spondylolisthesis”, and “controls”) were tested for with Bonferroni post hoc corrected ANOVA. All numerical data processing and statistical analysis were performed on Excel (Excel for Microsoft 365, Microsoft Corporation, Redmond, WA, USA) and SPSS (IBM SPSS Statistics 27, IBM, Armonk, NY, USA). A *p*-value below 0.05 was considered statistically significant.

## 3. Results

The demographic characteristics of the study groups are listed in Table 1. Individuals of the “degenerative” group were significantly older compared with the remainder (73.5 ± 11.1 years, *p* < 0.01). The mean ages in the “spondylolysis” and “controls” groups were 55.5 ± 15.7 years and 54.8 ± 15.7 years, respectively.

The severity of spondylolisthesis was noted as up to Meyerding grade 3 in the “spondylolysis” cases and a maximum of grade 2 in “degenerative”. For the vast majority of cases in either “spondylolysis” or “degenerative spondylolisthesis” groups, abnormal findings were identified in the fifth lumbar vertebra and/or involving the level between the fifth lumbar vertebra and the sacrum (Table 2).

Across the two readers, the “Darth Vader sign” was reported in 26–32 of 40 “spondylolysis” images, 0–1 of 13 cases with “degenerative spondylolisthesis”, and in 1 (both readers) of 86 “controls”. The inter-reader agreement between R3 and R4 with regard to the presence or absence of spondylolysis defined by the “Darth Vader sign” was substantial (κ = 0.77; 95% confidence interval: 0.64–0.90).

With respect to subgroups (all cases vs. bilateral spondylolysis only), the diagnostic performance of the “Darth Vader sign” was different between readers.

For analysis of all spondylolysis cases, the least experienced resident reader (R3) performed with a sensitivity of 65% and an almost perfect specificity of 99%. In comparison, the R4 reader had a markedly higher sensitivity of 80% at a specificity level of 98%. For bilateral spondylolysis cases only, the diagnostic performance was better with sensitivity and specificity values of 77% and 99% vs. 88% and 96% (R3 vs. R4), respectively. In summary, sensitivity and specificity ranged between 65.0–88.2% and 96.2–99.0%, respectively (Table 3).

Diagnostic performance for the detection of spondylolysis using the “Darth Vader sign” was excellent, with comparable AUC values for both readers of 0.89 and 0.82, respectively. The accuracy was slightly higher for the detection of bilateral spondylolysis cases, with excellent and outstanding accuracy (AUC of 0.878 and 0.922 for R3 and R4, respectively).

## 4. Discussion

This study serves to introduce a newly identified sign, named the “Darth Vader sign”, into the body of radiological literature. The “Darth Vader sign” has shown a substantial level of inter-reader agreement, as well as demonstrated excellent accuracy for the diagnosis of lumbar spondylolysis on routinely acquired CT images of the abdomen and pelvis that cover the spine. We believe that this newfound sign can be easily taught and subsequently implemented during the reporting routine, thereby improving the detection of spondylolysis in CT imaging.

The diagnostic accuracy reported in this study concurs with the conclusion drawn by Teplick [14], who proved that the majority of spondylolysis cases can be reliably identified in regular axial images without the need for multiplanar reconstructions. Furthermore, our observations are in line with the existing literature regarding the anatomic site of spondylolysis, as, generally, pars interarticularis defects tend to primarily occur in the fifth lumbar vertebra [14].

Our investigation takes a spot in a series of efforts that have been undertaken to improve the detection rate of spondylolysis across different modalities. The most commonly known qualitative sign has been described by Millard in 1976, who identified “The Scotty dog in his collar” on lateral radiographs of the lumbar spine [40]. Moreover, Talangbayan made an uncommon observation and recognized the shape of a well-known emperor in cases of spondylolysis, which led to the subsequent description of the “Inverted Napoleon Hat sign” for standard radiographic examinations of the lumbar spine in routine anterior–posterior or posterior–anterior projection [41]. Radiography is still often used as the primary imaging modality for the workup of suspected spondylolysis and/or spondylolisthesis [22]. However, there has been a significant increase in the utilization of CT scanners over the past decades [42,43].

Recent clinical guidelines have recommended considering CT imaging in patients who exhibit symptoms suggestive of spondylolysis and/or spondylolisthesis, along with inconclusive conventional radiography or physical examination [22]. Furthermore, CT is recommended as the imaging modality of choice in elite athletes with lower-back pain and edema of the pars interarticularis [44]. However, there exists literature supporting the belief that spondylolysis and other skeletal diseases may be underreported when non-skeletal reading of body CT scans are performed [32,33,34]. Moreover, during our investigation, we discovered that, in our very own situation, there were less than 100 study-eligible patients, while looking over a period of 4 years, which is suggestive of a significant degree of underreporting and/or inconsistency in terminology at our own institution. Hence, promoting the recognition of spondylolysis in CT, especially when not performed for skeletal purposes, might have a certain value. In cross-sectional imaging, a “wide canal” is known as a qualitative image finding of spondylolysis. However, to identify this sign, sagittal reformations are required, and the pars interarticularis defect itself has to be accompanied by a certain degree of spondylolisthesis to fulfill the criteria of a “wide canal sign” [45].

In contrast, the implementation of the “Darth Vader sign” in routine reporting requires no additional reconstruction or windowing and would thus allow for seamless integration into a non-skeletal reading routine without additional effort. Our study did not investigate sophisticated methodological or technological advances in clinical imaging. However, we are convinced that memorable and easy-to-use diagnostic ancillaries can help with improving diagnostic detection rates in general and are crucial to be adequately proven with regard to usability and diagnostic accuracy. As an example, at our institution, clinicians commonly order radiographs for spondylolysis assessment. However, in the majority of patients, prior CT imaging of the torso would be available and allow us to exclude defects of the pars interarticularis without an additional examination. It is likely that referring physicians are not aware of this fact, since spondylolysis is rarely mentioned in reports of non-skeletal body CTs. With the introduction of the “Darth Vader sign” into the literature, we aim to raise awareness of the need for second looks at pre-existing (cross-sectional) image data prior to ordering new examinations. This may help in reducing overall radiation exposure and could contribute to cost effectiveness related to diagnostic radiology.

Given recent developments in artificial intelligence and its utilization in clinical imaging, we might assume that our sign could be reported automatically as an incidental finding using the appropriate image analysis algorithms. The spectrum of artificial intelligence applications in academic and clinical musculoskeletal radiology is continuing to evolve, and algorithms are known to perform particularly well in high-volume, low-complexity tasks. The identification of spondylolysis would likely require two-step algorithms, with the first being necessary for lumbar level detection. The second algorithm may be based on pattern recognition in single axial images. Both of these tasks in spinal imaging have been described recently [46].

It is important to acknowledge the following limitations of our study. First, we included cases based on written reports as ground truth, which is susceptible to a relevant selection bias. However, we believe that the impact on the validity of our results is negligible, as we did not aim to assess the prevalence of the pathology in this study. Second, our ground truth was based on expert opinion, and we did not investigate the diagnostic performance of the “Darth Vader sign” in comparison with other diagnostic features and signs. However, we are not aware of any broadly accepted qualitative or (semi-) quantitative imaging parameters for the interpretation of spondylolysis and therefore believe that a pure visual assessment by an expert is appropriate as the reference standard. Finally, we did not evaluate the clinical relevance of lumbar spondylolysis in patients.

## 5. Conclusions

In conclusion, the “Darth Vader sign” shows excellent diagnostic performance at a substantial inter-reader agreement for the detection of spondylolysis in standard axial reconstructions of the trunk covering the lumbar spine. Implementation of this important Force into radiology residency curriculums may help in improving detection rates and subsequently contribute to a better understanding and robust data with regard to the prevalence of this condition among asymptomatic individuals.

## Figures and Tables

**Table 1:** Patient demographics.

Group	Spondylolysis	Degenerative Spondylolisthesis	Controls
Cases (*N* = 59)	40 (68%)	13 (22%)	6 (10%)
Age (mean ± SD)	55.5 ± 15.7	73.5 ± 11.1 *	54.8 ± 15.7
Sex (%)	14 (35%) Female	8 (62%) Female	2 (33%) Female
Meyerding Grade (0/1/2/3/4)	9/13/13/5/-	-/11/2/-/-	-

Asterisk (*) indicates significant difference compared with other groups (*p* < 0.01). SD = Standard deviation.

**Table 2:** Frequency and anatomic level of spondylolysis and spondylolisthesis.

Spondylolysis		Degenerative Spondylolisthesis	
L 3	2	L 3/4	1
L 4	4	L 4/5	5
L 5	34 (6 unilateral)	L 5/S 1	7
Overall	40 (6 unilateral)	Overall	13

L = Lumbar vertebral level. S = Sacral vertebral level.

**Table 3:** Diagnostic performance of the “Darth Vader sign”.

	All Spondylolysis Cases		Bilateral Spondylolysis Cases	
Reader	R3	R4	R3	R4
Sensitivity (%)	65	80	76.5	88.2
Specificity (%)	99	98	99	96.2
AUC	0.820	0.890	0.878	0.922

Data are presented separately per reader. R3 = 3rd year resident. R4 = 4th year resident. AUC = Area under the receiver operating characteristic curve.

## Data Availability

The data presented in this study are available on request from the corresponding author.

## Abbreviations

ANOVA	Analysis of variance
AUC	Area under the curve
CT	Computed tomography
PACS	Picture archiving and communication system
